# Revisiting the Sham: Is It all Smoke and Mirrors?

**DOI:** 10.1093/ecam/neq074

**Published:** 2011-04-14

**Authors:** Brandon Horn, Judith Balk, Jeffrey I. Gold

**Affiliations:** ^1^Eastern Center for Complementary Medicine, PC, Los Angeles, California, USA; ^2^American University of Complementary Medicine, Los Angeles, California, USA; ^3^Semel Institute for Neuroscience and Human Behavior, University of California Los Angeles, Los Angeles, California, USA; ^4^Department of Anesthesiology Critical Care Medicine, Childrens Hospital Los Angeles, Los Angeles, California, USA; ^5^Department of Obstetrics and Gynecology, University of Pittsburgh School of Medicine, Los Angeles, California, USA; ^6^Departments of Anesthesiology and Pediatrics, Keck School of Medicine, University of Southern California, Los Angeles, California, USA

## Abstract

The misuse of sham controls in examining the efficacy or effectiveness of Complementary and Alternative Medicine has created numerous problems. The theoretical justification for incorporating a sham is questionable. The sham does not improve our control of bias and leads to relativistic data that, in most instances, has no appropriate interpretation with regards to treatment efficacy. Even the concept of a sham or placebo control in an efficacy trial is inherently paradoxical. Therefore, it is prudent to re-examine how we view sham controls in the context of medical research. Extreme caution should be used in giving weight to any sham-controlled study claiming to establish efficacy or safety.

## 1. Background

The gold standard of clinical trials is the Randomized Controlled Trial (RCT) [1], with the placebo control as one of the primary tools to achieve meaningful outcomes. In exploring the efficacy or effectiveness (hereafter collectively referred to as “efficacy”) of Complementary and Alternative Medicine (CAM) therapies, it has been assumed that similar standards should be applied. This article challenges the conventional assumption that placebo or sham controls can and should be used to establish the efficacy of CAM or other therapies. Specifically, it addresses the misuse and abuse of these study designs throughout the medical literature. Given the inherent paradox of even the concept of a placebo or sham control, this article argues that such controls should rarely, if ever, be used in clinical trials designed to establish whether a given treatment is efficacious. The purpose of the Sham control is given in the following.

### 1.1. A Question of Quality

Research into medical procedures can be divided into four broad questions: (i) Relative safety: how likely is the procedure to cause more harm than the disease it is designed to treat? (ii) Efficacy: does a given medical procedure create a beneficial change? (iii) Mechanisms: how does a given medical procedure produce this change? and (iv) Economics: how does it compare to other available treatments with respect to risks, benefits, safety and effectiveness? Establishing the relative safety of an intervention is of paramount importance. However, there is a range of considerations when determining the safety for a particular intervention, which is beyond the scope of the current article. Nonetheless, for research on human subjects, safety should be established prior to evaluating other outcomes. Thereafter, research can begin to focus on efficacy. If an intervention is not found to be efficacious, then committing resources to examine the mechanisms of action or the economics is not warranted. Historically, sham and placebo controls (hereafter collectively referred to as “sham” controls) have been identified as a useful methodological considerations to examine the efficacy of a given intervention. Over the past 50 years sham controls have been used in pharmacological and non-pharmacological study designs to establish the efficacy of an active intervention. However, with very few exceptions, sham-controlled trials have numerous limitations, are poorly executed and improperly interpreted. The current article details reasons why sham controls are scientifically inappropriate study designs when examining questions of intervention efficacy.

### 1.2. Sham Controls and Efficacy

Efficacy is defined as the extent to which a specific intervention, procedure, regimen or service produces a beneficial result under ideal conditions [2]. Where one is attempting to establish the efficacy of a given procedure, the study design must be crafted in a way that has the potential to provide an unequivocal answer. A sham control has been broadly defined as “a treatment or procedure that is performed as a control and that is similar to, but omits a key therapeutic element of the treatment or procedure under investigation” [3]. Commonly, sham controls are implemented because they are believed to reduce bias by controlling for the ancillary effects of a procedure [4]. In many instances, particularly in CAM research, shams are designed to control for psychological effects in an attempt to examine what is believed to be the active part of an intervention [5, 6]. For example, in a study looking at the efficacy of acupuncture for the treatment of low back pain, a treatment group received acupuncture at acupuncture points that were hypothesized to actively treat the pain. A second group received sham acupuncture. In this group, participants received actual acupuncture; however, the needles were inserted into acupuncture points that were not believed to actively treat the pain. By comparing these two groups, researchers were able to control for psychological components of the treatment, such as expectation of a benefit. It is noteworthy that this particular model is often based on unproven assumptions that placing needles in alternate or “sham” points will not in any way affect the specific outcome measure (in this case, low back pain).

## 2. Does a Sham Actually Control for Bias?

The use of a sham requires several assumptions. Importantly, it presumes that the psychological aspects of a given procedure are ancillary to the procedure. However, this has never been established. To the contrary, there is mounting evidence that psychological effects may in fact be relatively unique and, therefore, non-ancillary aspects of any given intervention. In an interesting study, Kaptchuk and colleagues at Harvard University [7] compared two different forms of placebo: sham acupuncture and a sham pharmaceutical pill (aka placebo pill) and they assessed the effects on headaches. As both a placebo pill and a sham technique are presumed to be inert, it was surprising that sham acupuncture was significantly more effective at relieving headaches than the sham pill. This and other studies demonstrating the wide range of effects that various placebos and shams can have on a given outcome measure strongly question the underlying assumption that psychological effects are distinct entities from any given intervention. If a psychological component of an intervention improves outcomes, but is relatively specific to that intervention, then it may not be appropriate to try to remove that component in an efficacy trial [8]. As such, it is a mistake to assume that psychological factors are ancillary to a given procedure *de facto* [9]. Consequently, any assumption a researcher makes that psychological factors are ancillary introduces bias, because this assumption has never been found to be accurate. While using a sham to control for psychological effects may remove some of the patient bias, it introduces an experimenter bias. Ultimately, we merely substitute one bias for another and the aggregation of bias is not improved.

## 3. Does a Sham Improve the Quality of Results in Efficacy Studies?

The next question is whether controlling for subject expectation improves the quality and value of the findings from efficacy studies. In many sham studies, the treatment procedure is altered to prevent the subject from being able to differentiate an active treatment from a sham treatment. While this improves the blinding of patients, it does so at the expense of obtaining an accurate assessment of the interventions potential efficacy. The contrived blinding technique often alters the actual conditions in which patients are being treated in the real world. As a result, one is studying a procedure that is not in clinical use. The results of the study, therefore, are not necessarily applicable to the procedure the researchers originally intended to study. For example, in acupuncture research, one of the most often-used sham needles requires the use of a relatively large plastic device, which alters the actual feel of the needle to both the practitioner and the patient. While this has been shown to effectively blind subjects, it also alters the practitioner's dexterity in needle manipulation [10]. Therefore, because the use of sham devices oftentimes reflects neither ideal conditions nor standard clinical conditions, we do not get quality data on whether the procedure is efficacious or effective. In some cases, there may be procedures and shams that can exactly duplicate real world scenarios and avoid the aforementioned pitfalls. For example, it could be that the ritual of taking a certain kind of sham pill may be no different than taking an active pill. However, even in such a case, the introduction of a sham to an efficacy trial creates fatal flaws.

## 4. Fatal Flaws: Smoke and Mirrors

Imagine the following scenario: Researchers attempt to examine the efficacy of acupuncture for improving pregnancy outcomes. To do so, a simple randomized, sham-controlled trial is set up comparing true acupuncture to sham acupuncture. In this case, the only difference between the active group and the sham group is the selection of acupuncture points. As such, subject bias is not an issue. Furthermore, the active treatment has not been altered by the use of a sham needle; therefore the active intervention is representative of actual clinical care. However, we now have a more severe issue. In this scenario, the results of the study showed that the acupuncture group achieved a 60% live birth rate and the sham group achieved a 50% live birth rate. The difference was not statistically significant. What conclusions can be drawn? The only conclusion one can properly make from this study is there is no significant difference between those two acupuncture point combinations. One cannot conclude anything at all about the efficacy of acupuncture, which was the stated objective of the study. The fatal flaw of this, and any other sham-controlled study that does not include a non-treatment control group or properly validated sham [6], is that the study is completely relativistic. There is no way of knowing if the findings are a false positive, false no-effect or false negative.

**Figure 1:**
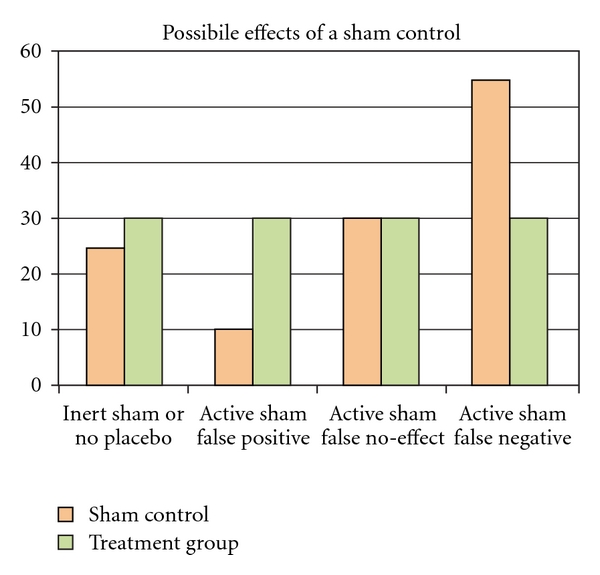
Possible effects of a sham control.

Consider the following illustration (Figure 1):

There are four possible scenarios for any simple sham-controlled study. The first possibility is that the sham truly has no effect on the outcome measure. In such a case, the sham is inert and, as such, is equivalent to having no placebo at all. The second possibility is that the sham has a negative effect on the outcome measure. This results in a false positive, where the actual treatment looks more effective than it really is. The third scenario is that the sham has a slightly positive effect on the outcome measure. In such a case, it appears that the actual treatment has done nothing. The fourth scenario is that the sham has a substantial positive effect on the outcome measure. In such a case, the actual treatment artificially appears to have a negative effect on the outcome. One of the above scenarios is always present in any sham-controlled study. Unless there exists either a non-treatment control group as an additional arm, or a sham that has been validated against the investigated outcome measure (which is exceedingly rare), it is impossible to know which scenario is occurring. As a result, the findings of most sham-controlled studies fail to provide data on the true effects of a given treatment. Study analyses and conclusions, however, rarely acknowledge this and frequently make unwarranted conclusions about the efficacy of a procedure [11], resulting in the discarding of valid procedures and the acceptance of procedures that may not be efficacious.

One unfortunate area where this confusion is perpetuated is in the establishment of safety data. For example, the New England Journal of Medicine (NEJM) published a study on the quadrivalent HPV vaccine (Gardasil) [12], examining its efficacy and safety. The researchers compared the vaccine to a sham vaccine (placebo). The sham, however, contained aluminum compounds that are known to cause adverse reactions [13, 14]. The researchers concluded that:

*There were relatively few side effects of vaccination* [emphasis added]. The proportion of subjects who reported one or more injection-site adverse events was higher in the vaccine group than in the placebo group (84.4% versus 77.9%) … One subject in the placebo group discontinued participation owing to a serious injection-related adverse event (hypersensitivity). The proportions of women reporting serious adverse events were similar in the two treatment groups … [N]o safety concerns among non-pregnant women were identified [in this trial] [12].

Clearly, concluding that a vaccine is safe based on such a trial design is grossly misleading and irresponsible. Because the vaccine appears to have the same high level of adverse events as the placebo does not mean that the vaccine is thus safe.

## 5. A Placebo Paradox: The Inherent Inconsistencies of Sham-Controlled Efficacy Trials

**Figure 2:**
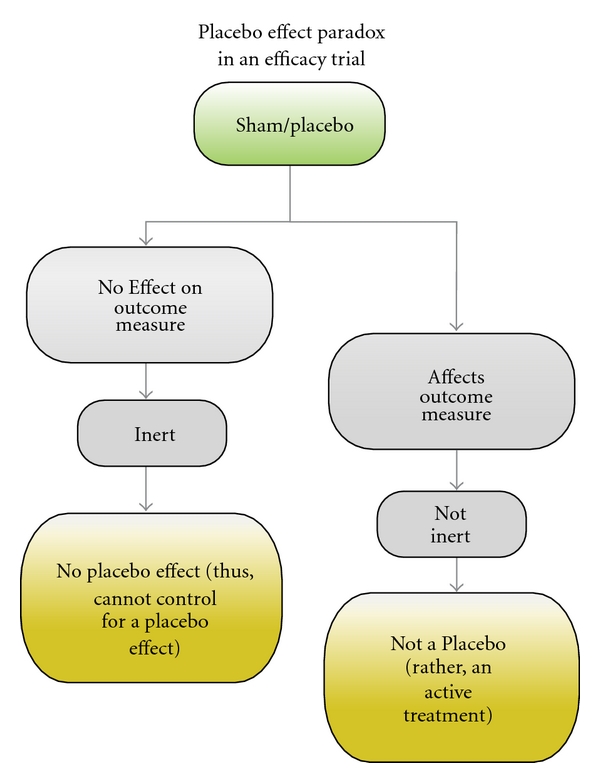
Placebo effect paradox in an efficacy trial.

As the Gardasil trial demonstrates, shams are not necessarily inert. However, a placebo or sham is supposed to be “a substance or procedure… that is *objectively* without specific activity for the condition being treated” [15]. Objectivity means that it must be validated through a controlled trial demonstrating no effect on the outcome measure. In the NEJM study, there was no mention that the sham had been objectively studied to have no effect on safety or on cervical cancer. This leads us to a paradox: one cannot use a sham that shows an effect on the outcome measure because it is not “without specific activity for the condition being treated”. In other words, it's not inert. For example, if a proposed sham for a study on pregnancy rates increases pregnancy rates, it is an active treatment and therefore one cannot use it as a “sham”. On the other hand, if a sham objectively does not show any effect on the outcome measure then, while it is inert, there obviously is no “placebo effect”. The whole reason for a placebo control study design is to control for a hypothesized placebo effect. In other words, if the control has no effect on the outcome measure, then there is no placebo effect either. If the control has no placebo effect, it is no different than using a non-treatment control (Figure 2). In such a case, it would waste resources that could potentially be used to increase the power of the study [10].

## 6. Conclusion

In conclusion, study design needs to be re-examined in light of modern medical knowledge. Sham studies cannot be used to establish efficacy except under one of two conditions. The first condition is that a third arm is used in the study that contains a non-treatment control or a validated sham. The second is that the sham must be validated against the outcome measure being examined. However, of note, a validated sham needs to be inert, without an effect on the outcome measure and therefore without a placebo effect. Thus, it should not be used in efficacy trials as it wastes resources that could be used to improve the power of the trial. The idea that controlling for the placebo effect is necessary for a quality efficacy study is, in part, based on outdated concepts that psychological factors are generic and non-unique. This has never been proven and, in fact, more recent studies are suggesting the contrary. Therefore, it is of no benefit in efficacy trials to try to control for any effects that can potentially only be achieved through the particular procedure being examined.

In reality, shams- and placebo-controlled trials are merely comparative trials. Once efficacy is established for a given modality, controlling for various aspects of a procedure can give us useful information regarding the mechanisms by which such procedure produces its beneficial results. This can be important in further refining and improving treatments. However, it is irrational to conduct a study to examine which aspects of a procedure are responsible for its benefit, if such benefit has not first been established. It is therefore prudent to re-examine how we view sham controls in the context of medical research. While they can be useful in the context of a comparative trial, extreme caution should be used in giving weight to any sham controlled study claiming to establish efficacy or safety.
